# Evaluation of a protective effect of in ovo delivered *Campylobacter jejuni* OMVs

**DOI:** 10.1007/s00253-016-7699-x

**Published:** 2016-07-06

**Authors:** Renata Godlewska, Maciej Kuczkowski, Agnieszka Wyszyńska, Joanna Klim, Katarzyna Derlatka, Anna Woźniak-Biel, Elżbieta K. Jagusztyn-Krynicka

**Affiliations:** 1 Department of Bacterial Genetics, Institute of Microbiology, Faculty of Biology, University of Warsaw, Miecznikowa 1, 02-096 Warsaw, Poland; 2 Department of Epizootiology and Clinic for Birds and Exotic Animals, Faculty of Veterinary Medicine, Wrocław University of Environmental and Life Sciences, Pl. Grunwaldzki 45, 50-366 Wrocław, Poland

**Keywords:** *Campylobacter jejuni*, Outer membrane vesicles (OMV), in ovo immunization

## Abstract

*Campylobacter jejuni* is the most prevalent cause of a food-borne gastroenteritis in the developed world, with poultry being the main source of infection. *Campylobacter jejuni*, like other Gram-negative bacteria, constitutively releases outer membrane vesicles (OMVs). OMVs are highly immunogenic, can be taken up by mammalian cells, and are easily modifiable by recombinant engineering. We have tested their usefulness for an oral (in ovo) vaccination of chickens. Four groups of 18-day-old chicken embryos (164 animals) underwent injection of wt *C. jejuni* OMVs or modified OMVs or PBS into the amniotic fluid. The OMVs modifications relied on overexpression of either a complete wt *cjaA* gene or the C20A mutant that relocates to the periplasm. Fourteen days post-hatch chicks were orally challenged with live *C. jejuni* strain. Cecum colonization parameters were analyzed by two-way ANOVA with Tukey post-hoc test. The wtOMVs and OMVs with wtCjaA overexpression were found to confer significant protection of chicken against *C. jejuni* (*p* = 0.03 and *p* = 0.013, respectively) in comparison to PBS controls and are promising candidates for further in ovo vaccine development.

**Electronic supplementary material:**

The online version of this article (doi:10.1007/s00253-016-7699-x) contains supplementary material, which is available to authorized users.

## Introduction

*Campylobacter* strains are recognized as a major causal agent of bacterial diarrhea both in developing and developed regions of the world. *Campylobacter* infection rates have been increasing steadily over the past decade. In EU countries, the number of confirmed human campylobacteriosis cases was 236,851 in 2014, an increase of 22,067 cases (9.6 %) compared to 2013 (EFSA and ECDC 2015). Mortality associated with *Campylobacter* infections is relatively low, and no specific treatment is required for a vast majority of patients. However, *Campylobacter* infections constitute a serious problem due to the high number of cases, severity of possible neurological complications, as well as high social and economic costs of the disease (Kaakoush et al. 2015; Kirkpatrick and Tribble 2011).

The consumption of infected poultry meat is a major source of infection. European Food Safety Authority (EFSA) reported that in 2014, 38.4 % of the 6703 samples of fresh broiler meat were found to be *Campylobacter* positive (EFSA and ECDC 2015). Efforts to comply with EU hygiene and biosecurity regulations appear insufficient to control or eliminate *Campylobacter* from the poultry food chain (Havelaar et al. 2007; Mangen et al. 2007). Elimination of *Campylobacter jejuni* from chickens would significantly reduce the incidence of campylobacteriosis in humans and seems to be an alternative and more realistic approach for controlling *Campylobacter* contamination. However, anti-*Campylobacter* chicken vaccines are not commercially available yet.

Many Gram-negative bacteria, especially pathogenic ones (such as *Neisseria meningitidis*, *Haemophilus influenzae*, *Helicobacter pylori*, *Borrelia burgdorferi*, *Pseudomonas aeruginosa*, *Shewanella* spp., *Shigella* spp., *Salmonella* spp.), produce outer membrane vesicles (OMVs). Secretion of these vesicles to a medium is a naturally occurring phenomenon. Vesicles contain not only outer membrane-associated proteins but also periplasmic and even cytoplasm-located molecules (virulence factors, DNA chains, enzymes) (Kuehn and Kesty 2005).

Considering their immunogenic and self-adjuvant properties, the ability to be taken up by mammalian cells, and adjustability of their content by recombinant engineering, OMVs are attractive candidates for vaccine delivery vectors. Composition of the OMVs (LPS, glycerophospholipids, OM and periplasmic proteins) makes them capable of mediating both pro- and anti-inflammatory activities leading to a clearance of infection or a widespread inflammation (Mashburn-Warren et al. 2008; Mashburn and Whiteley 2005). That is why OMVs are useful as vaccines and adjuvants stimulating protective mucosal and humoral immune responses (Collins 2011), already constituting a base of several licensed vaccines and gaining an increasing popularity (van der Pol et al. 2015). For example, conventional wild-type outer membrane vesicle (wtOMV) vaccines are the only formulations that have shown efficacy against serogroup B meningococcal disease (Acevedo et al. 2014; Asensio et al. 2011; Holst et al. 2009; Roberts et al. 2008). Consequently, a novel vaccine (4CMenB), against serogroup B meningococcal disease, composed of protein antigens identified by reverse vaccinology (fHBP fused to GNA2091, GNA2132 fused to GNA1030, and NadA), combined with OMVs is now approved in Europe, Canada, Australia, and some Latin American countries (Carter 2013; Giuliani et al. 2006; Martin and Snape 2013; Serruto et al. 2010).

*Campylobacter jejuni* OMVs have not yet attracted a comparable interest. It has only been shown that *C. jejuni* membrane vesicles contain a toxin-dubbed cytolethal distending toxin (CDT) (Elmi et al. 2012; Lindmark et al. 2009), and proteomic analyses of *C. jejuni* OMVs identified 151 proteins, including periplasmic, outer membrane associated, inner-membrane, and even cytoplasmic ones. Among them, all three subunits of CDT (CdtA, CdtB, and CdtC) and sixteen-glycosylated proteins were present (Elmi et al. 2012; Jang et al. 2014).

One of the latter is CjaA, an extracytoplasmic, glycosylated and highly immunogenic protein cloned and characterized in our laboratory (Pawelec et al. 1997; Wyszynska et al. 2008). CjaA is conserved between different *Campylobacter* serotypes. Crystallographic analyses of the *Escherichia coli*-produced rCjaA determined that CjaA binds to the cysteine ligand and is a constituent of the ABC cysteine transport system (Muller et al. 2005). Expression of CjaA in bacteria cultured on solid media increases under iron deficiency conditions and is higher in clinical *Campylobacter* isolates than in laboratory strains (Cordwell et al. 2008; Holmes et al. 2005; Shoaf-Sweeney et al. 2008). These facts suggest that CjaA participates in a colonization process in vivo.

CjaA is recognized by chicken maternal antibodies (Cordwell et al. 2008; Shoaf-Sweeney et al. 2008), and several groups attempted to utilize it as an antigen for immunization of chickens. We were first to show that vaccination with live *S. enterica* sv. Typhimurium χ3987-expressing CjaA reduces the colonization with heterologous *C. jejuni* strain by as much as six logs (Wyszynska et al. 2004). Oral immunization of SPF birds with *S. enterica* sv. Typhimurium *aroA* mutant producing CjaA antigen fused to the C-terminal part of the fragment C of tetanus toxin resulted in about 1.4 log10 CFU/g reduction in the cecal load of *C. jejuni* (Buckley et al. 2010). Similarly, *Salmonella*-mediated delivery of the linear epitope of CjaA fused to the LamB protein and coexpressed with the immuneenhancing CD154 ligand caused approximately 2-log reduction in ileal load of *C. jejuni* (Layton et al. 2011). Finally, oral inoculation of SPF chicken with *Eimeria tenella* oocysts, modified to express CjaA on the surface, induced 91 and 86 % immune protection against *C. jejuni* challenge compared with unvaccinated and wild-type *E. tenella*-vaccinated controls (Clark et al. 2012). All these experiments demonstrated a promising, albeit not sufficient protection; importantly, the effect was dependent also on chicken breeds used.

In this study, we employed yet another delivery mode and evaluated the protective effect of a nonadjuvanted, multivalent OMV vaccine administered in ovo against infection with *C. jejuni*, employing natural OMVs as well as OMVs enriched with CjaA due to the introduction of an extra copy of the wt *cjaA* gene or C20A mutant of the *cjaA* known to shift the protein localization into periplasm. Our work demonstrated that the immunization of chickens with OMVs provides a mean to reduce a cecal colonization with wt *C. jejuni.*

## Materials and methods

### Bacterial strains, media, and culture conditions

Bacterial strains and plasmids used in this study are listed in Table 1.

**Table 1:** Strains and plasmids used in this study

Strain or plasmid	Genotype/resistance/description	Reference
*Campylobacter jejuni*		
81176 (ATCC BAA-2151)	Wild type, human isolate	Korlath et al. (1985)
81176 *cjaA* ^−^	pVir, *cjaA*::*aphA3*, Tc^R^	This study
12 (PCM 2852)	Wild type; isolated from a chicken, good colonizer	Wyszynska et al. (2004)
12/2	Wild type; isolated from a chicken with introduced pUOA18 plasmid; good colonizer, Cm^R^	(Wyszynska et al. 2004)
*Escherichia coli*		
*E. coli* S17.1	F‾ *rec*A *thi pro hsd*R^−^ M^+^ RP4:2-Tc:Mu:Km Tn7 Tp^R^ Sm^R^ λpir Tra^+^	Simon et al. (1983)
Plasmids		
pUWM639	*cjaA* gene (from 81176) cloned into pRY111, Cm^R^	This study
pUWM1405	*cjaA* gene (from 81176) with C20A point mutation cloned into pRY111, Cm^R^	This study

*Campylobacter jejuni* strains were grown on Blood Agar no. 2 (BA, Oxoid) plates supplemented with 5 % horse blood, *Campylobacter* selective supplement (Blaser-Wang) (Oxoid, Basingstoke, UK), and tetracycline (10 μg/ml) at 37 °C or 42 °C for 16–24 h under microaerobic conditions (6 % O_2_, 10 % CO_2_, 85 % N_2_). If necessary, plates were also supplemented with chloramphenicol (20 μg/ml) and/or kanamycine (25 μg/ml). *Campylobacter* strains used for isolation OMVs were grown in Mueller–Hinton broth under microaerobic conditions at 37 °C.

*Campylobacter jejuni* 12/2 strain, isolated from an intestinal tract of a chicken, labeled with the pUOA18 plasmid containing *cat* gene, was utilized in challenge experiments.

### Site-directed mutagenesis

Point mutation was generated using the QuikChange site-directed mutagenesis kit according to procedures recommended by the supplier (Stratagene). A derivative of pBluescript II SK, containing the *cjaA* gene with its own promoter from *C. jejuni* 81176 pVir, was used as a template for PCR-mediated mutagenesis. Point mutation C20A was introduced with primers CAGTAGTATTGGCTGCTGCTGGAGGAAATTCTGACTCTAAAAC and GTTTTAGAG TCAGAATTTCCTCCAGCAGCAGCCAATACTACTG (mismatches are double underlined).

The plasmid containing the *cjaA* gene with C20A was transformed into *E. coli* TG1, and a presence of the desired mutation was verified by DNA sequencing. After digestion of the resulting recombinant plasmid with *Not*I and *Sal*I, the fragment containing the *cjaA* gene was cloned into the shuttle vector pRY111 digested with the same enzymes. The resulting plasmid was named pUWM1405 (*cjaA* with C20A mutation). Shuttle plasmid containing a wild copy of the *C. jejunicjaA* gene was named pUWM639. All derivatives of pRY111 were introduced into *C. jejuni cjaA*^−^ by bi-parental conjugation (Davis et al. 2008).

We had to recreate the *C. jejuni* 81176 *cjaA*^−^ mutant. The one used in our previous studies had lost pTet plasmid, which could be important in conjugative transfer of plasmids. New mutant was constructed using standard natural transformation assay (Vegge et al. 2012). Exponentially, growing cells of *C. jejuni* 81176 Tc^R^ were transformed with genomic DNA of *C. jejuni* 81176::CjaA^−^, Tc^S^ strain. The disruption of *cjaA* gene was confirmed by PCR and sequencing.

### OMV purification and quantitation

OMVs were isolated from *C. jejuni* strain 81176 using methods described by Elmi et al. (2012) and Chutkan et al. (2013).

*Campylobacter jejuni* 81176 strain was grown in the Mueller–Hinton broth under microaerobic conditions at 37 °C. An overnight culture was diluted 1:100 into 330 ml of fresh growth media and grown 16–18 h to a mid log phase. Briefly, the cells were pelleted twice, using centrifugation at 6000×*g* for 2 × 20 min at 4 °C. Supernatants were filtered through a 0.22-μm filter device to remove remaining cells. The filtrate was ultracentrifuged in Beckman L7-55 Ultracentrifuge at 150,000×*g* for 3 h at 4 °C, using a 50.2 Ti rotor. OMV preparations were plated on BA plates and incubated under microaerobic conditions to confirm the absence of viable bacteria.

The pellets were resuspended with Dulbecco’s phosphate-buffered saline (DPBS) or PBS and stored at −20 °C until needed. Before injecting into eggs, the OMV samples were sterilized by filtration using Ultrafree-MC-GV Centrifugal filters (Merck Millipore). A protein content of OMVs was measured by BCA assay.

### Enrichment of *C. jejuni* OMVs with the CjaA protein

Plasmid pUWM639, carrying an additional copy of *cjaA* gene on the shuttle vector (pRY 111 plasmid), was introduced into 81176 *C. jejuni cjaA*^−^ strain by standard bi-parental conjugation procedure (with *E. coli* S17.1 strain) (Davis et al. 2008). Similarly, pUWM1405, with C20A point mutation in *cjaA* gene, was introduced into 81176 *C. jejuni cjaA*^−^ to increase the CjaA content in periplasm. The OMVs produced by *C. jejuni* 81176/pUWM639 and *C. jejuni* 81176/pUWM1405 strains were purified as described above. The quantification of CjaA protein in OMV from abovementioned strains was carried out using enzyme-linked immunosorbent assay (ELISA). Serial dilutions of total, heat-treated (100 °C for 20 min) OMVs were used to coat 96-well plates. The recombinant CjaA antigen was used as the plate-coating antigen for construction of a standard curve. Coated wells were incubated with specific rabbit anti-rCjaA antibodies and with alkaline phosphatase-conjugated second goat anti-rabbit IgG antibodies (Sigma-Aldrich). The reaction was run with *p*-nitrophenyl phosphate (1 mg/ml) as a substrate and was stopped after 30-min incubation (room temperature) with 3.0-M sodium hydroxide. Optical density was determined at 405 nm using the ELISA reader (Tekan). Each sample was analyzed in triplicate. The results were expressed relative to the standard protein per milligram of total OMV protein.

### Animal supply and housing

Animal experiments were performed using the Rosa1 breed of chickens. This breed was created by crossing Sussex hen with Rhode Island Red rooster. The birds were housed in separate cages for each group and given water and food ad libitum. All animal experiments were carried out with approval nr 397/2012 of the LKE (First Local Ethical Committee on Animal Testing in Warsaw).

### Immunization and challenge regimen

One hundred sixty-four 18-day-old chick embryos of laying hens (Rosa1 breed) were orally immunized by injection of 0.1-ml different OMVs (wtOMVs, 639-OMVs, 1405-OMVs) into the amniotic fluid. Each injection dose contained 200 μg of protein. Groups of birds inoculated with PBS buffer served as controls. Fourteen-day post-hatch half of all chicks groups were orally challenged with 10^5^-CFU of the live *C. jejuni* 12/pUOA18 strain. Cloacal swabs were taken every 2 days after hatch to evaluate *Campylobacter* colonization of birds. After the challenge, the *C. jejuni* present in chicken cecal contents were enumerated by plating. On 21 and 28 days after hatch, chicks were sacrificed, and their cecal contents were aseptically removed. Samples were weighed and nine times their weight of PBS was added. Samples were homogenized, and serially diluted. 0.1 ml of each dilution was plated on *Campylobacter* BA plates, supplemented with chloramphenicol. The scheme of the protection experiment is shown in Table 2.

**Table 2:** Scheme of immune response and protection experiments

Week of life	−1	0	1	2	3	4
Immunization with OMVs	+					
Collection of gut secretion and blood samples for immune response analysis		+	+	+	+	+
Challenge with *Campylobacter*				+		
Cecum isolation for *Campylobacter* enumeration					+	+

Samples of intestinal content and blood were collected on 2, 7, 14, 21 and 28-day post-hatch as shown in Table 2. ELISA test was performed to determine the level of *C. jejuni*-specific immunoglobulin Y (IgY) antibodies and immunoglobulin A (IgA) in serum and intestinal secretion samples.

### ELISA for serum IgG antibodies and intestinal IgA antibodies

The level of antibodies against *C.jejuni* protein in intestinal secretions and sera of chicken was quantified by ELISA.

Ninety-six-well plates (Nunc, Thermo Scientific) were coated overnight at 4 °C with whole-cell *C. jejuni* proteins (20 μg/well), washed three times with PBS containing 0.02 % Tween 20 (Sigma-Aldrich), blocked for 1 h at 37 °C with PBS containing 2 % bovine serum albumin (Sigma-Aldrich), washed as described previously, and incubated for 1.5 h at room temperature with either diluted sera (1:300) or intestinal secretion samples (1:10). The plates were developed with 3,3′,5,5′-tetramethylbenzidine (Sigma-Aldrich), using goat anti-chicken IgA horseradish peroxidase conjugate (Thermo Scientific, Rockford, IL) or rabbit anti-chicken IgY (whole molecule)-peroxidase (Sigma-Aldrich) (dilution 1:2000), respectively. The plates were incubated with the substrate for 25 min at room temperature and then the colorimetric reaction was stopped by adding 2-M H_2_SO_4_ (Sigma-Aldrich). Absorbance was measured at 490 nm using an ELISA reader (Tekan). Each sample was analyzed in duplicate.

### Statistical analyses

All statistical analyses of the colonization results were performed using GraphPad Prism 6 (GraphPad Software, San Diego, CA) or STATISTICA 10PL software (StatSoft, USA). The significance of differences between the obtained values was appraised using one-way analysis of variance (ANOVA), followed by the post-hoc Tukey’s tests. Statistical analyses of ELISA test were assessed using multifactorial (two-way) ANOVA nested for duplicates and Scheffe’s post-hoc test or the Kruskal–Wallis test followed by Dunn’s multiple comparison post-hoc test. Any *p* values <0.05 were considered significant.

## Results

### Enrichment of *C. jejuni* OMVs with the CjaA protein

We decided to utilize OMVs for in ovo immunization of chickens, additionally attempting to enrich OMVs with extra CjaA in order to increase the load of the antigen in the vaccine. Two strategies were employed toward this goal. The first one relied on expressing an additional copy of the complete *cjaA* gene introduced into the multicopy plasmid shuttle vector pRY111 (Yao et al. 1993). The constructed plasmid was named pUWM639. Since *cjaA* expression from pUWM639 was driven by its own strong promoter, it seemed plausible that this manipulation would increase the content of CjaA protein inside the cells and within OMVs. The second strategy took advantage of the fact that CjaA in *Campylobacter* cells localizes mainly in the inner membrane (IM). We had previously created a *Campylobacter coli* 72Dz/92 strain with the C20A (cysteine into alanine) point mutation in the CjaA signal sequence. Lack of this cysteine shifted the location of the protein from inner membrane to periplasm (Wyszynska et al. 2008). We expected that the periplasmic location of CjaA will further increase the quantity of this protein in OMVs. Toward this end, the pUWM1405 plasmid was prepared that contained the C20A *cjaA* mutant introduced into pRY111.

Both pUWM639 and pUWM1405 plasmids were introduced into *C. jejuni* 81176 *cjaA*^−^ strain via conjugation. To ensure an easy detection of the introduced gene expression, the host strain had been previously deprived of endogenous CjaA protein by means of a gene knockout. Western blot experiments with specific rabbit anti-rCjaA antibodies showed a high level of protein production (Fig. S1, supplementary materials).

**Fig. 1:**
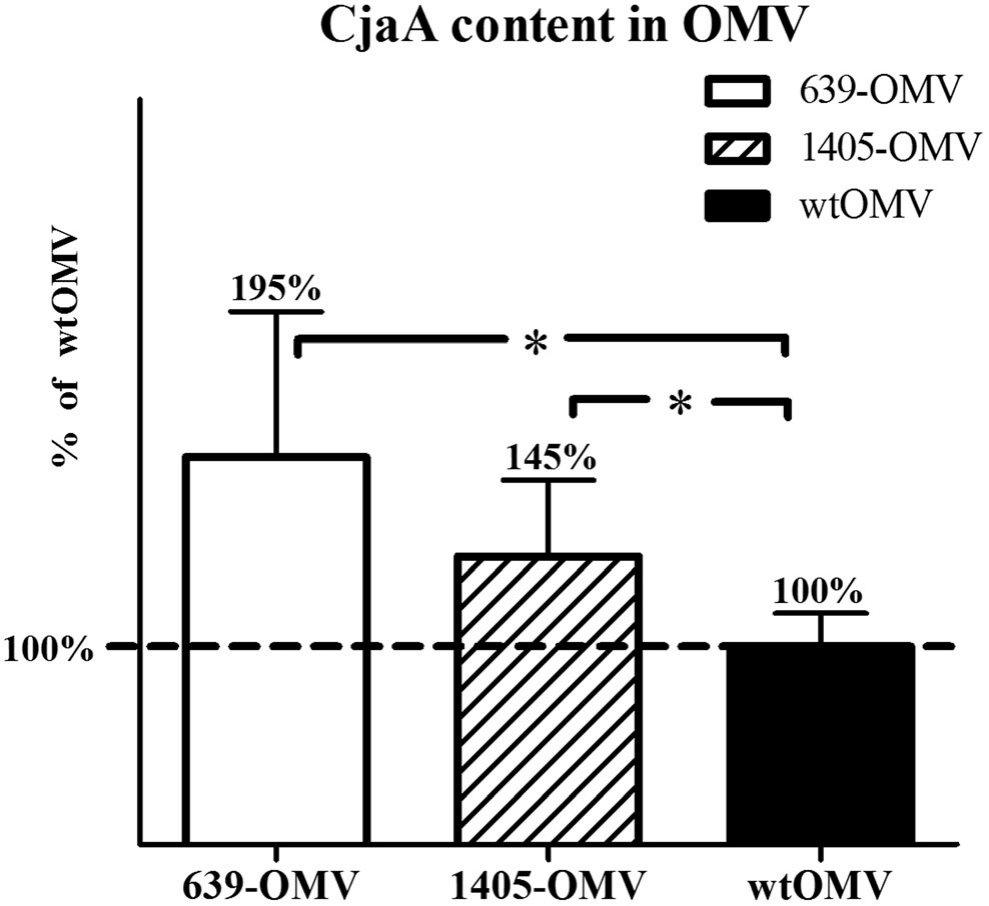
Comparison of protein CjaA content in different types of OMVs. CjaA in wtOMV was marked as 100 %. The quantification of CjaA protein in OMV-639, OMV-1405, and wtOMV was carried out using ELISA assay. Serial dilutions of total OMVs were used to coat 96-well plates. The recombinant CjaA antigen was used as the plate-coating antigen for construction of a standard curve. Coated wells were incubated with specific rabbit anti-rCjaA antibodies and with alkaline phosphatase-conjugated second goat anti-rabbit IgG antibodies. Each sample was analyzed in triplicate. The results were expressed relative to the standard protein per milligram of total OMV protein. A statistical analysis was carried out using multifactorial (one-way) ANOVA followed by Tukey’s multiple comparisons test. *Asterisks* indicate significant differences (*p* < 0.05) between analyzed groups and the control group

ELISA assay of the CjaA protein in OMVs isolated from wt strain and 81176 *C. jejuni* strains harboring pUWM639, and pUWM1405 plasmids demonstrated that OMVs produced by modified strains harbored much higher a content of CjaA antigen than wt OMVs. The results are shown in Fig. 1.

### Immunization procedure. Antibody immune response in vaccinated chickens

A multiantigenic nature of an OMV vaccine may enhance specific humoral immune response to provide protection against *C. jejuni* strains. Therefore, the antibody response of chicks to OMVs delivered in ovo was analyzed. Eighteen-day-old embryos were divided into four groups (one control and three treatment groups) and orally immunized by OMVs injection into an amniotic fluid. The control group received sterile PBS. The treatment groups were immunized with wtOMV, 639-OMV, or 1405-OMV. Several experimental studies have shown that in commercial flocks, chicks are colonized by *C. jejuni* around second or third week of life (Conlan et al. 2007). Therefore, 2 weeks after hatching, half of the chicks from each group were challenged with 10^5^ bacterial cells of a chicken-isolated *C. jejuni* strain. Serum IgY and mucosal IgA antibody responses against *Campylobacter* proteins were measured by ELISA assay using the whole-cell lysate as a coating antigen. Analysis of serum samples obtained from 1-, 7- to 14-day-old chicks showed relatively high levels of specific IgY in all groups, which dramatically decreased by week 2. Starting at 1 week after challenge, anti-*Campylobacter* IgY antibody level consistently increased in all experimental groups (data not shown). IgY antibody titers did not differ significantly among the four experimental groups at any of the time points (Kruskal–Wallis test).

**Fig. 2:**
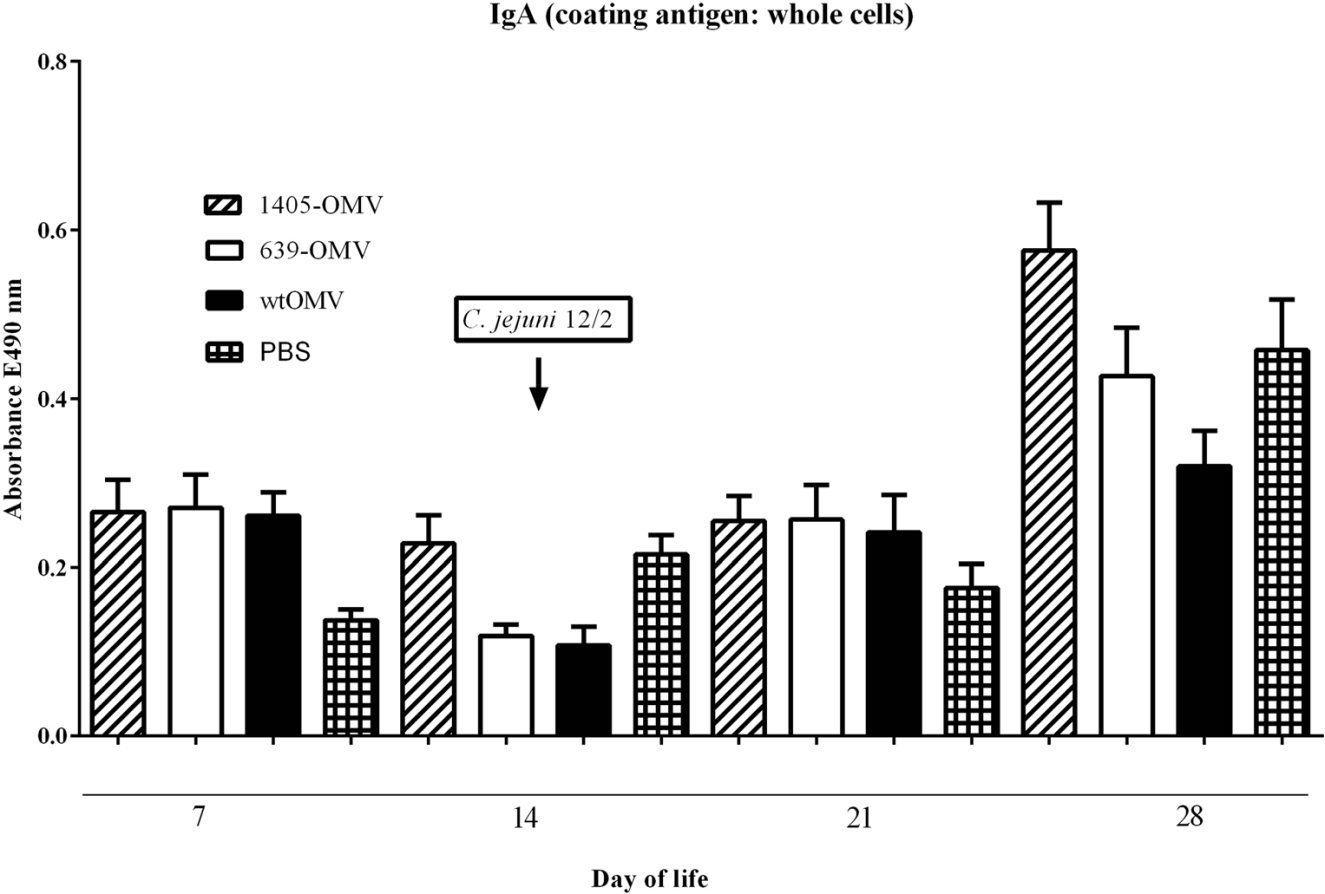
Mean anti-*Campylobacter*-specific mucosal IgA levels measured by ELISA at the time intervals on the x-axis. Various types of OMVs were administred in ovo to 18-day-old chickens embryos. Intestinal samples (ceca) were collected, when birds were 1, 2, 3, and 4 weeks old. *Campylobacter* whole-cell lysates were used as coating antigens. Intestinal secretion samples were diluted 1:10. *Error bars* indicate standard error of the mean. Statistical analyses of ELISA test were assessed using multifactorial (two-way) ANOVA followed Scheffe’s post-hoc test. Statistical analyses were performed using STATISTICA 10PL software (StatSoft, USA). Any *p* values <0.05 were considered significant

Analysis of the intestinal samples also showed an induction of specific mucosal anti-*Campylobacter* IgA antibodies (Fig. 2). We observed an increasing level of IgA antibody 2 weeks after challenge (28 days of life), but the differences again were not statistically significant.

Multifactorial (two-way) ANOVA nested for duplicates of ELISA measurement indicated that IgA levels in challenged animals significantly correlated with time and OMVs type (*p* < 0.00001 and *p* < 0.0009, respectively), and there was also a significant interaction of time and OMV type (*p* < 0.004). Overall, there was a very significant increase of IgA at day 28 (*p* < 0.000001) and a slight decrease at day 14 compared to day 7 (*p* = 0.024; Scheffe’s post-hoc tests). Overexpression of a C20A mutant of CjaA was the only manipulation significantly affecting IgA level, leading to a slight increase in comparison to PBS and wt treatment (*p* < 0.02 and *p* < 0.004, respectively, Scheffe’s post-hoc test) in univariate analysis; for interactions, expression of a C20A mutant led to a very significant increase of IgA at day 28 comparing to most other combinations, other changes were nonsignificant or meaningless at best.

### Protective efficacy of OMVs in ovo immunization of chickens

**Fig. 3:**
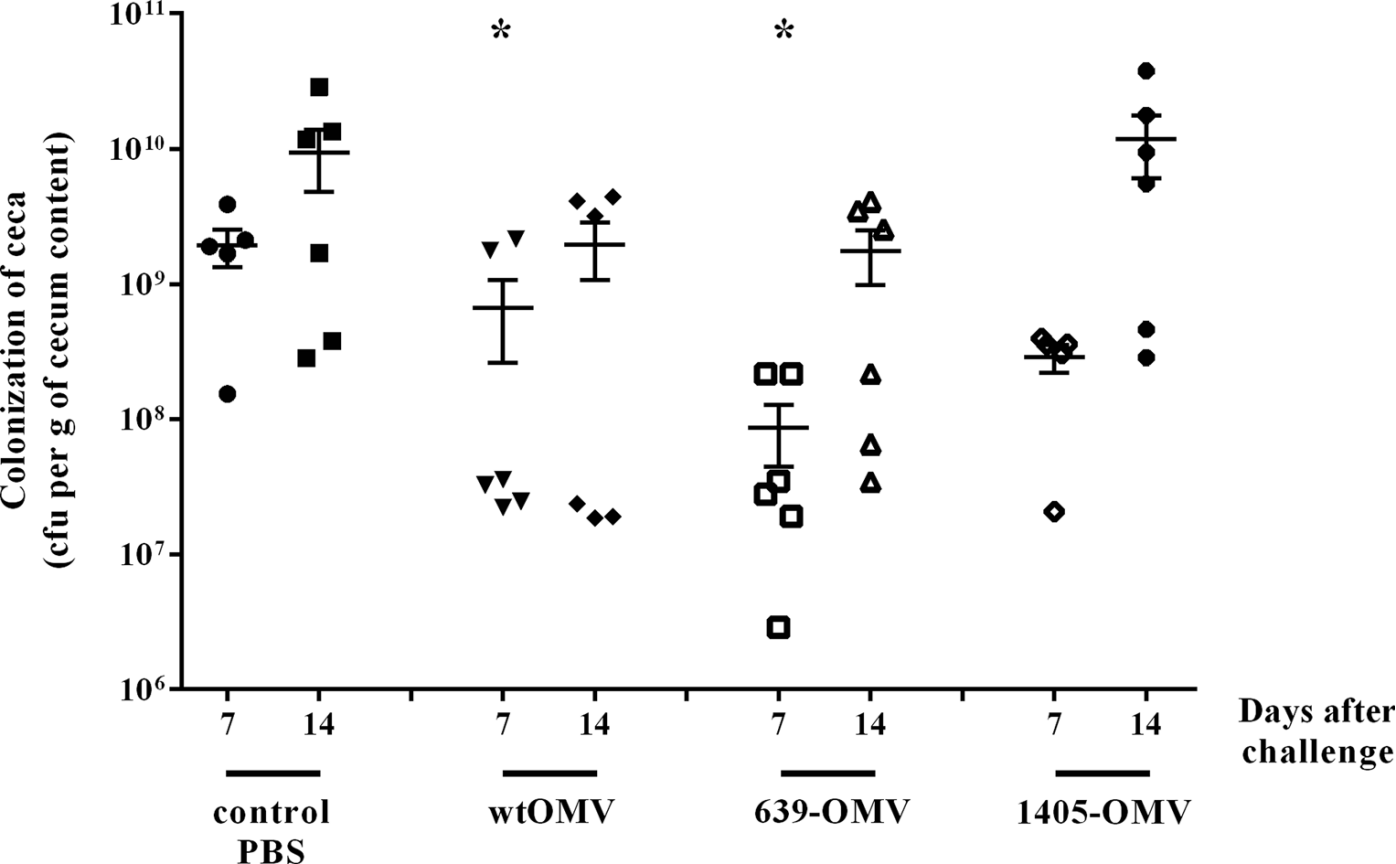
Colonization of chickens vaccinated with *C. jejuni* OMVs: wtOMV, 639-OMV, and 1405-OMV after *C. jejuni* challenge. Eighteen-day-old chicken embryos were given one dose of OMVs and 2 weeks after hatching was challenged with *C. jejuni* 12/2. Control birds were given PBS. Viable *C. jejuni* cells were recovered from the ceca of chickens 7 and 14 days post-infection (d.p.i.). Bacterial recoveries represent colonization levels of five or six birds per time interval. A geometric mean for each group is denoted by a *bar*. A statistical analysis was carried out using multifactorial (two-way) ANOVA followed by Tukey’s multiple comparisons test. *Asterisks* indicate significant differences (*p* < 0.05) between analyzed groups and the control group

Three different types of OMVs, namely wtOMVs, 639-OMVs, and 1405-OMVs were tested for protective effect in Rosa1 chickens. In this study, 18-day-old chicken embryos were in ovo vaccinated with a single dose of OMVs vaccine by oral routes and challenged 2 weeks after hatch with the heterologous wild-type strain marked by plasmid pUOA18. As shown in Fig. 3, the in ovo immunization with 10^5^-CFU/ml of *C. jejuni* strain (12/2) provided a moderate protection against *Campylobacter* infection at the seventh day after challenge in two vaccinated groups (wtOMV and 639-OMV, *p* < 0.05). Our results indicated that mean colonization levels were of 2 × 10^9^ CFU in control group and from ∼9 × 10^7^ in 639-OMV group to 3 × 10^8^ in wtOMV and 1405-OMV groups. Two weeks after challenge, the colonization level of immunized bird’s ceca was still lower than nonimmunized bird’s ceca, but the results for immunized groups were not statistically significant (Fig. 3).

Multifactorial (two-way) ANOVA on log-transformed CFU data demonstrated that both the time from the challenge and the type of OMVs were independently correlating to colonization level (*p* < 004 and *p* < 0.007, respectively). The impact of time indicates an increasing level of infection after 2 weeks compared to 1-week post-challenge. The impact of vaccination with OMVs was further analyzed post-hoc using Tukey’s test, revealing that OMVs from wt strain or strain overexpressing wtCjaA confer significant protection.

There was no indication of significant interactions between the time from the challenge and the type of OMV used (*p* > 0.44).

## Discussion

Diverse strategies have been applied to produce an effective anti-*Campylobacter* vaccine for chickens. The greatest progress has been made in the development of subunit vaccines composed of *Campylobacter* proteins, whether delivered directly or by vectors such as live-attenuated *Salmonella* cells expressing *Campylobacter* antigens (Buckley et al. 2010; Layton et al. 2011; Wyszynska et al. 2004). However, the real-life results of their usage are mediocre at best, and new approaches are clearly needed.

The major goal of this study was, therefore, to analyze the potential of OMVs derived from *C. jejuni* as a new vaccine compound. Here, for the first time, we combined OMVs as a carrier of antigens with in ovo vaccination as a delivery mode to obtain an alternative method for post-hatch immunization of chickens against *C. jejuni*.

In ovo antigen delivery stimulates both innate and adaptive immune responses and can be used for vaccination (especially against viral diseases), for beneficial modification of the bacterial profile in the colon of chickens, for stimulation of an immunological response and embryonic development, and for testing of teratogenic effects (Bande et al. 2015; Negash et al. 2004; Toro et al. 2007).

The avian immune system begins to develop early during embryogenesis, and antibody responses to antigens could be induced in 12-to-14-day-old chicken embryos (Sharma 1999). However, a full immunocompetence normally builds up only after few days of post-hatching (Mast and Goddeeris 1999). Earlier on, the immune defense is provided by maternal antibodies, which had been transferred from a hen to offsprings through egg yolk. Thus, the few days after hatching are crucial: chicks become exposed to environmental threats but are no longer supplemented with new maternal antibodies. And although the gut-associated lymphoid tissue (GALT), which provides an important enteric protection, matures functionally around fourth day after hatching, the secretory IgA response against enteric antigens mounts slowly, completing toward the end of the second week of age (Bar-Shira et al. 2003).

The in ovo vaccination can fill this gap, generating immunity as early as possible, even by the time of hatch.

In order to further improve the in ovo vaccination, we have established and employed a simple way to enrich OMVs with the CjaA antigen based on simple recombinant DNA techniques. We believe that this method is applicable to many other antigens.

Since the protective role of antibodies is regarded as a key factor in the development of poultry vaccines against *Campylobacter*, we investigated the IgY and IgA levels in chicken from control and immunized groups. Our results are consistent with earlier reports. Sera of chickens contained large quantities of maternal IgY during the first week of life, which decreased gradually during next 2 weeks and resumed to rise after a colonization of the intestinal tract by *C. jejuni.* However, IgY antibodies play little role in the clearance of *C. jejuni* from cecum, a decrease in the number of *C. jejuni* organisms colonizing the intestinal tract has been observed (Newell and Fearnley 2003; Sahin et al. 2003; Shoaf-Sweeney et al. 2008).

Mucosal IgA is responsible for preventing entry of commensal bacteria into subepithelial areas by blocking their adhesion to epithelial cells or by returning those bacteria that have already penetrated to the basolateral site, without eliciting an inflammatory response (Brisbin et al. 2008). They probably play an important role in redirecting inflammatory response toward tolerance. As in the case of IgY, we observed increasing levels of IgA antibodies starting at 1 week after *C. jejuni* challenge, which proves that *Campylobacter* challenge triggers birds immune response. In both experiments, the level of mucosal IgA and IgY in serum of noninfected was lower compared to colonized chicken.

Immunization with wtOMVs and OMVs enriched in the CjaA antigen had a significant effect on the cecal load of *C. jejuni* 7 days after challenge, when compared to vaccination with control preparation. Fourteen days after challenge, the *C. jejuni* cecal loads were still reduced; however, the differences were not statistically significant anymore. It is puzzling why the periplasmic mutant of CjaA (1405) failed to confer a protection comparable to ones obtained with OMVs from wt and 639 strains. We have verified (Fig. 1) that the expression of this mutant in OMVs was actually higher than wt protein (albeit lower than overexpressed wt protein). This result was obtained using ELISA with polyvalent anti-CjaA rabbit serum. It is possible that the membrane protein, forcefully relocated (released) to periplasm, does not retain (or does not achieve) its normal conformation and/or modifications. Regarding the latter, this mutant remains to appear as a double band on western blotting, which is demonstrated to reflect the glycosylation that it is normally subjected to (Wyszynska et al. 2008). However, this does not preclude that the glycosylation is incomplete/atypical or that the protein is otherwise altered, thus having a distorted antigenicity. As a result, it would be less efficiently detected in ELISA by a polyvalent serum raised against wt protein (leading to false impression of ‘decreased’ level comparing to overexpressed wt protein), but more importantly, it would elicit an immune response shifted toward ‘false’ antigens, thus offering decreased protection against wt strains presenting only a typical wt CjaA. This explanation remains speculative as it was not further investigated.

Thus, a single vaccination regime for 18-day-old embryos has induced a protection against *C. jejuni* challenge, but the protection was too short lived. Efforts should be undertaken to enhance OMV vaccine efficacy by combining OMV with appropriate adjuvants or additional protein antigens like in 4CMenB in anti-*N. meningitidis* vaccine (Carter 2013; Martin and Snape 2013). Nevertheless, most of the recently tested vaccines required two doses for a full protection. Thus, the additional dose of a vaccine administrated after hatch may still be needed.

The time and route of administration of such a second dose deserve consideration. Presumably, the booster dose should be given when the defense system is well developed, which is about 7 days after hatch (Mast and Goddeeris 1999).

Regarding the route, in ovo vaccines are typically injected into the amniotic sac. The embryo then swallows and ingests surrounding amnion fluid containing vaccine antigens. Antigens subsequently pass into the intestine, where they can stimulate the immunocompetent cells of GALT (Jochemsen and Jeurissen 2002).

Orally administrated vaccine must, therefore, survive a passage through the crop and stomach into the intestine. Protein antigens combined with OMV vaccines may thus benefit from an additional protection, e.g., liposome encapsulation.

It is not certain whether the booster should follow the same route. Several studies have tested various administration routes of OMV vaccines in other animals. Schild et al. (2008) immunized mice with OMVs derived from *Vibrio cholerae* via three routes (intranasal, intragastric, and intraperitoneal). Interestingly, all the routes studied had comparably strong effect on Ig titers (IgG1, IgG2, IgM, and IgA were studied) and provided comparable protection against *V. cholerae* infection and colonization. The intranasal and intragastric immunized mice were fully protected, and from intraperitoneal immunized mice, at least 10,000-fold lower numbers of *Vibrio* CFU were recovered than in the infection of naïve animals. Nieves et al. (2011) demonstrated that a subcutaneous immunization with OMVs isolated from *Burkholderia pseudomallei* provides a significant protection against lethal *B. pseudomallei* infection in BALB/c mice.

Thus, the oral route for booster in chicken may be preferred as the simplest one, but further studies are needed to verify and compare its efficacy to other modes of delivery.

In conclusion, we presented a proof-of-concept study on the usability of *C. jejuni* OMVs enriched with CjaA antigen as an in ovo protective vaccine against *C. jejuni* in chickens. While the response after single dose of a vaccine is clearly observable, the effect was rather short lived. Further studies on booster dosing and/or immunoadjuvant support are required.

## Electronic supplementary material

ESM 1 (PDF 339 kb)
