# Supplementary material for: Evaluation of a protective effect of in ovo delivered Campylobacter jejuni OMVs
Source: Appl Microbiol Biotechnol. 2016 Jul 6;100(20):8855–64. doi: 10.1007/s00253-016-7699-x (PMC5035662; doi:10.1007/s00253-016-7699-x)
Supplement: Supplementary file 1 — (PDF 339 kb) [file 253_2016_7699_MOESM1_ESM.pdf]

Supplementary material

Applied Microbiology and Biotechnology

Evaluation of a protective effect of *in ovo* delivered *Campylobacter jejuni* OMVs

Renata Godlewska[1]*, Maciej Kuczkowski[2], Agnieszka Wyszy ska[1], Joanna Klim[2], Katarzyna Derlatka[1], Anna Wo niak-Biel[2], El bieta K. Jagusztyn-Krynicka[1]

[1] Department of Bacterial Genetics, Institute of Microbiology, Faculty of Biology, University of Warsaw, Miecznikowa 1, 02-096 Warsaw, Poland

[2] Department of Epizootiology and Clinic for Birds and Exotic Animals, Faculty of Veterinary Medicine, Wrocław University of Environmental and Life Sciences, pl. Grunwaldzki 45, 50-366 Wrocław, Poland

*Corresponding author:
Renata Godlewska, renatag@biol.uw.edu.pl

Fig.S1

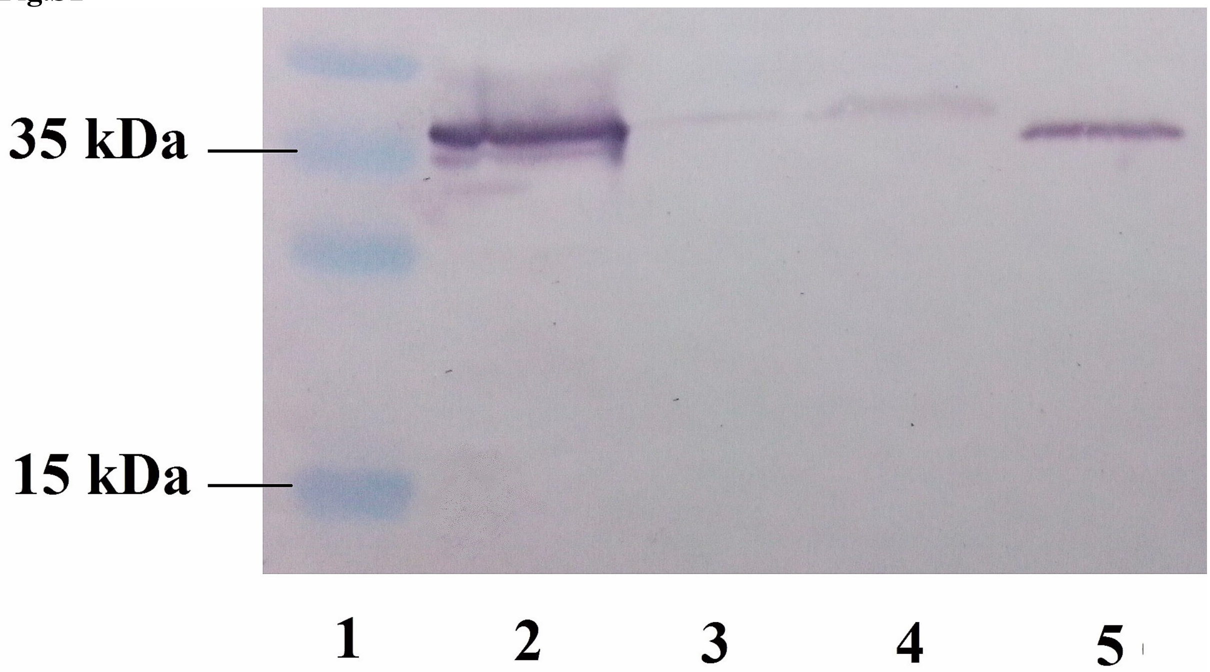

Fig.S1 Immunoblot analysis of proteins isolated from wild-type *C. jejuni* 81-176 and from the OMVs: wt-OMV, 1405-OMV, 639-OMV with rabbit antiserum against recombinant CjaAx6His. *Campylobacter* protein extracts were electrophoretically separated on a 12 % polyacrylamide gel, blotted onto nitrocellulose membrane and probed with polyclonal anti-rCjaA antibodies. The relative positions of molecular weight markers are listed on the left (in kilodaltons). Lanes: 1. molecular size standard; 2. *C. jejuni* 81-176 wt (whole cells); 3. wt-OMV; 4. 1405-OMV; 5. 639-OMV
